# Adaptive evolution and inherent tolerance to extreme thermal environments

**DOI:** 10.1186/1471-2148-10-75

**Published:** 2010-03-12

**Authors:** Jennifer Cox, Alyxandria M Schubert, Michael Travisano, Catherine Putonti

**Affiliations:** 1 Department of Biology, Loyola University Chicago, Chicago, IL, USA; 2 Department of Bioinformatics, Loyola University Chicago, Chicago, IL, USA; 3 Department of Ecology, Evolution, and Behavior, University of Minnesota, St Paul, MN, USA; 4 Department of Computer Science, Loyola University Chicago, Chicago, IL, USA

## Abstract

**Background:**

When introduced to novel environments, the ability for a species to survive and rapidly proliferate corresponds with its adaptive potential. Of the many factors that can yield an environment inhospitable to foreign species, phenotypic response to variation in the thermal climate has been observed within a wide variety of species. Experimental evolution studies using bacteriophage model systems have been able to elucidate mutations, which may correspond with the ability of phage to survive modest increases/decreases in the temperature of their environment.

**Results:**

Phage ΦX174 was subjected to both elevated (50°C) and extreme (70°C+) temperatures for anywhere from a few hours to days. While no decline in the phage's fitness was detected when it was exposed to 50°C for a few hours, more extreme temperatures significantly impaired the phage; isolates that survived these heat treatments included the acquisition of several mutations within structural genes. As was expected, long-term treatment of elevated and extreme temperatures, ranging from 50-75°C, reduced the survival rate even more. Isolates which survived the initial treatment at 70°C for 24 or 48 hours exhibited a significantly greater tolerance to subsequent heat treatments.

**Conclusions:**

Using the model organism ΦX174, we have been able to study adaptive evolution on the molecular level under extreme thermal changes in the environment, which to-date had yet to be thoroughly examined. Under both acute and extended thermal selection, we were able to observe mutations that occurred in response to excessive external pressures independent of concurrently evolving hosts. Even though its host cannot tolerate extreme temperatures such as the ones tested here, this study confirms that ΦX174 is capable of survival.

## Background

Changes in environmental conditions alter the outcome of natural selection, affecting the selective benefits of subsequent adaptations that improve an organism's fitness in the new conditions. In the case of extreme environmental change, the appearance and fixation of beneficial adaptations can be essential for survival. A large number of different factors, e.g. resource availability, temperature, community dynamics, etc., can drive adaptive evolution. In particular, species' responses to climate changes are of growing concern, and numerous studies have been carried out for a wide variety of organisms in nature as well as the laboratory, e.g. pikas [1], fish [2], honey bees [3], daphnia [4] and microbes [5], amongst many others.

Within eukaryotic and even bacterial species, identifying the correspondence between minute molecular adaptations and changes in the organism's fitness is challenging. In contrast, bacteriophages have frequently been isolated from nature and used as model laboratory systems due to their much more compact genomes and the corresponding relative ease for assessing molecular responses to selection. Moreover, while each phage has a small genome, phages are the most abundant form of life on earth and play a pivotal role within the global ecosystem [6,7]. As such, numerous studies have been conducted examining phages and their thermal environment in nature. Metagenomic studies have examined the diversity of marine viruses, which includes phages, in diverse thermal conditions, from thermal vents and hot springs [8-10] to samples from the bitter cold of Antarctica [11,12].

In the laboratory, adaptive evolution experiments have been performed for individual phage species, exploring their viability at temperatures both within and exceeding their thermal niche [5,13-20]. In addition to assessing adaptations made in response to new selective pressures phenotypically (typically with respect to the survival rate and/or morphology of plaques), mutations occurring in response to a particular thermal environment can be directly determined and monitored over generations. The range of temperatures and duration of exposure differs from one study to another; moreover, the means in which the treatment is applied also varies. For instance, many of the studies of the phages ΦX174 and G4 have explored the response to elevated temperatures on the phage-host pair, in other words plating/culturing the phage and bacterial host (typically incubated at 37°C) at a range of temperatures: 42°C to 56°C [13], 38°C to 43.5°C [20], 37°C to 45°C [16], 41.5°C to 44°C [19], and 27°C to 44°C [5,17]. Despite the knowledge garnered from previous studies of *E. coli *growth at different temperatures [21-23], identifying the adaptations of the phage due solely from the thermal environment and not from a host response to the environment is challenging. Avoiding such obstacles, studies have also been conducted in which changes in temperature were applied to just the phage, e.g. ΦX174 [14], T4 [15], and Φ6 [18].

Thus far, research has chiefly focused on relatively modest changes in thermal environment. Survival and adaptive evolution to extremes remains largely unknown. Here, using the well-studied ssDNA bacteriophage ΦX174, elevated temperatures both for relatively short as well as long durations of time are explored. In contrast with other studies of temperature selection for this phage [5,13,16,17,19,20], thermal treatments are focused on the phage itself. Extreme temperatures, comparable to those occurring within hot springs (74°C +) [10], were explored to assess the ability of ΦX174 to survive and adapt in novel environments.

## Results

### Acute thermal selection

**Figure 1:**
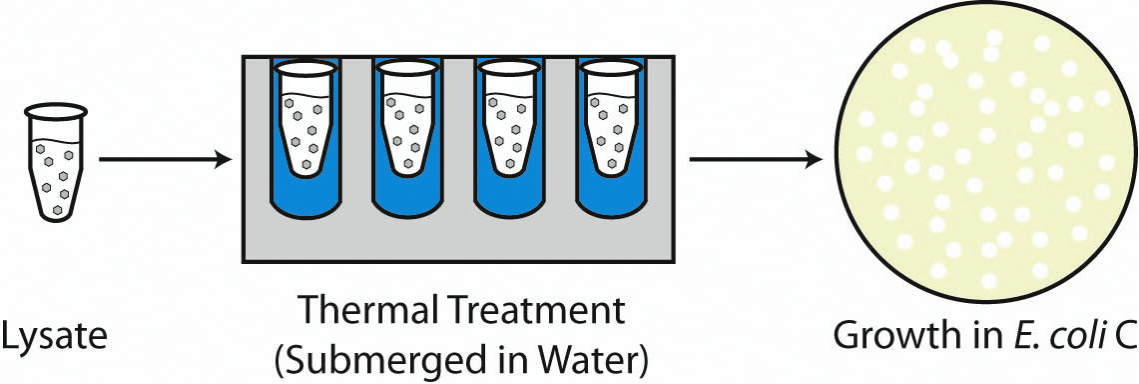
**Experimental design for short-term treatment of phage lysate to elevated temperatures**. Each virus lysate tube was submerged in water and treated at either 50°C or 70°C for 1, 2, 3, or 4 hours, after which it was plated with *E. coli *C.

**Figure 2:**
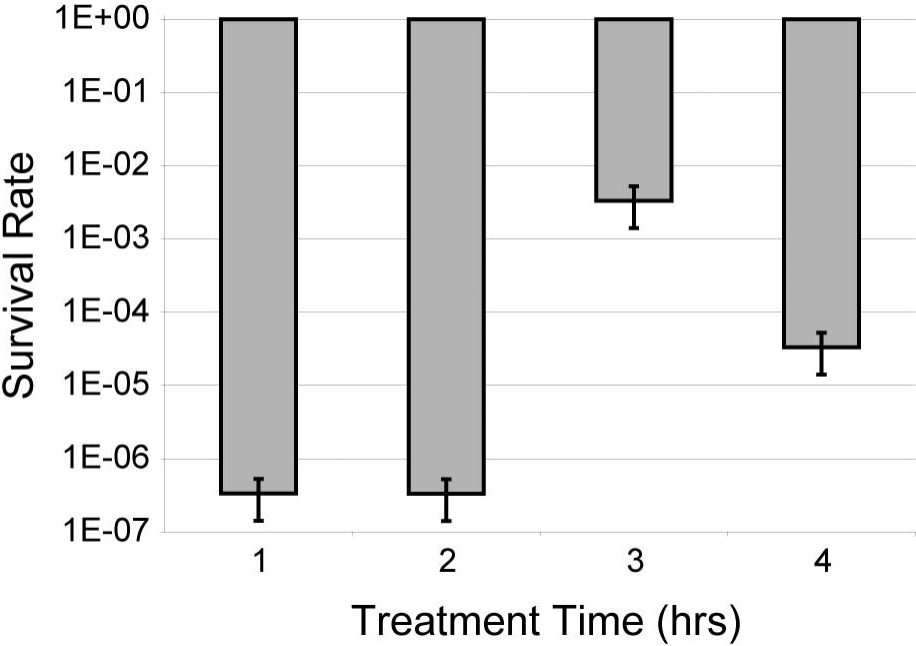
**The average survival rates and standard error of the mean for the 70°C treatments**.

Following the experimental design illustrated in Figure 1 (see Methods), plaque numbers for phage treated at 50°C, regardless of the time of exposure, were unchanged relative to control phage lysates; dilution series were carried out confirming a minimal (less than one order of magnitude) decline in phage survival (results not shown). In contrast, many of the samples subjected to 70°C did not form any plaques when plated. Comparison of the number of PFU of the control lineage, the Anc strain, (in essence time point 0) and each of the treatment lines allows us to calculate the average survival rate. Table 1 delineates the results of a two-way ANOVA of the survival rates for all 24 samples (12 treated at 50°C and 12 at 70°C) revealing that only temperature is found to be statistical significant. The average survival rate for the 50°C treatment samples was 98% and for the 70°C treatments was 0.0083%. The survival rate within the 70°C treatments varied according to the exposure time; the average survival rates and standard error of the mean for the 70°C treatments are shown in Figure 2. It is important to note, however, that only two-thirds of phage populations at the 70°C treatment temperature survived. The number of plaques produced was drastically smaller than that of the 50°C treatments.

**Table 1:** Two-way ANOVA results for short-term heat treated isolates.

Source of Variation	SS	df	MS	F	P-value
Temperature	5.989902	1	5.989902	1437433	4.63E-41
Time	1.24E-05	3	4.14E-06	0.993199	0.42113
Interaction	1.24E-05	3	4.14E-06	0.993199	0.42113

Plaques from eight of these 70°C treatment plates were isolated and their adsorption rate was quantified (see Methods, refr. [24]). Table 2 lists the adsorption rates for the heated (70°C) isolates as well as the untreated isolate (time point 0). While two of the eight acute selected isolates had higher rates of adsorption than the control, the majority exhibit a reduced adsorption rate.

**Table 2:** Adsorption rates for short-term heat (70°C) treated isolates.

Duration of Selection	Adsorption Rate (ml/min)
Untreated Control	1.94 × 10^-10^
1 hour	1.40 × 10^-10^
1 hour	7.06 × 10^-10^
2 hours	4.80 × 10^-11^
2 hours	8.60 × 10^-10^
3 hours	1.67 × 10^-9^
3 hours	2.00 × 10^-10^
3 hours	2.78 × 10^-10^
4 hours	8.56 × 10^-10^

As previous studies suggest [16,19], the ability to endure this elevated temperature is likely due to adaptations within the structural proteins. Thus, we sequenced a ~2700 bp region encompassing the structural coding regions of the isolated plaques to identify possible beneficial mutations; each sequenced region was compared to the ancestral strain's genomic sequence in order to identify what mutations occurred. The sequences of the isolates from the 50°C treatments were identical to the ancestor Anc sequence. On the contrary, the 70°C treatment isolate sequences included several mutations (Table 3): the E150D mutation occurred within the D protein coding region (also in the overlapping E coding region); the A45V, L242F and Q349H within the F protein coding region; and the V44L, T63S and G79V within the H protein coding region. All of the mutations observed were nonsynonymous; individual nucleotide changes are listed in Additional file 1: Supplemental Table S1. In total four different genotypes were observed. The average adsorption rates for each genotype are: 1.40 × 10^-10 ^(E150D/L242F/Q348H/V44L/T63S), 1.67 × 10^-9 ^(G79V), 1.75 × 10^-10 ^(E150D/A45V/L242F/Q349H/T63S), and 8.07 × 10^-10 ^(Q349H/T63S).

**Table 3:** Nonsynonymous mutations observed in the short-term heat (70°C) treated isolates.

	Protein Coding Region						
Duration of Selection	D	F			H		
1 hour	E150D		L242F	Q349H	V44L	T63S	
1 hour				Q349H		T63S	
2 hours	E150D	A45V	L242F	Q349H		T63S	
2 hours				Q349H		T63S	
3 hours							G79V
3 hours	E150D	A45V	L242F	Q349H		T63S	
3 hours	E150D	A45V	L242F	Q349H		T63S	
4 hours				Q349H		T63S	

### Extended thermal selection

**Figure 3:**
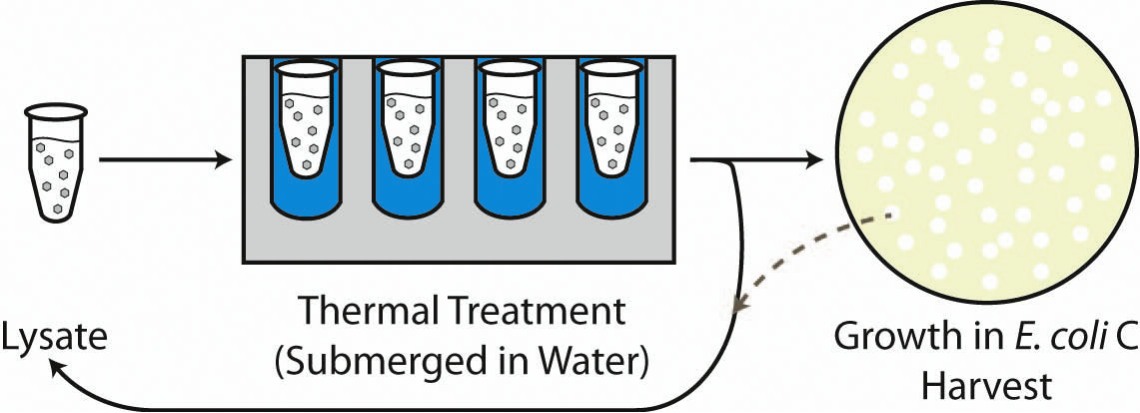
**Experimental design for long-term treatment of phage lysate to elevated temperatures**. Each virus lysate tube was submerged in water and heated, after which it was either entirely plated with *E. coli *C or an aliquot was plated and the remaining lysate was used for subsequent heating. In the case of the former, the plate was harvested after overnight incubation and used for subsequent treatments (as indicated in the figure by the gray dashed line).

We next explored the evolution of the phage to withstand longer exposure to elevated temperatures. In particular, we were interested in determining the extent of adaptation to 70°C resulting from extended selection. When creating a high titer ΦX174 stock for use in the extended thermal selection experiments, the population incorporated two nonsynonymous mutations (in the F and H coding region) resulting in a modified strain, which we refer to as JACS, serving as the ancestral population of the extended thermal selection experiments (see Methods). This high titer lysate was subjected to much longer durations of heat exposure, ranging from 24 to 96 hours (Figure 3 and Table 4). Just as in the acute thermal treatments, the mutations occurring within the phage during these treatments are a result solely of the temperature, as has been previously observed [15], rather than in response to the host or replication errors. After each selection period, 100 μl of lysate was sampled onto *E. coli *C while the remaining lysate solution was then returned to thermal selective conditions. Periodically, the entire lysate was plated on the host, and following overnight incubation, the plate was harvested and used for subsequent heat treatments (as indicated by the gray dashed arrow in Figure 3). These brief periods of relaxed selection, in the absence of elevated/extreme temperatures, were critical. As the number of viable phage decreases, the probability of their collection between heat treatments (and their formation of plaques when plated) also decreases. By plating the entire remaining lysate and allowing for a population expansion, we could readily assess the viability of the entire population and decrease the potential for population extinction. The observations made during these platings served as the rationale behind the times and temperatures of successive rounds of selection (see Methods). In addition, this also assisted in maintaining a relatively constant solution volume within the tube under treatment while allowing the treated phage of replicating within the host and the associated introduction of genetic variability.

**Table 4:** Long-term heat treatment protocol. Those rounds with an "x" for the Replating column signify that the entire treatment tube was plated on *E. coli *C and incubated overnight at 37°C.

Selection round	Temperature (°C)	Duration (hours)	Replating
1A (Line A)	70	24	
1B (Line B)	70	48	
2	50	24	x
3	75	72	x
4	70	24	x
5	50	65	
6	50	24	x
7	50	96	x
8	50	24	
9	50	72	x

Table 4 lists the temperature and duration of heat exposure for each sequential period of selection as well as the stages within the protocol for which the entire lysate was plated and harvested for subsequent treatment. The first treatment administered, of either 24 hours at 70°C (henceforth referred to as "Line A") or to 48 hours at 70°C ("Line B"), quickly culled all phages unable to withstand this elevated temperature; thus one my conclude that such phages accumulated irreparable mutations as a result of the long-term extreme temperature exposure which prohibited them from later infecting and reproducing within the host. In total, 18 replicates (nine for each line) were subjected to thermal selection ranging from 50°C to 75°C, lasting over 17 days in total. After each selection period, a sample of each lysate population was plated as well as titered (see Methods).

**Figure 4:**
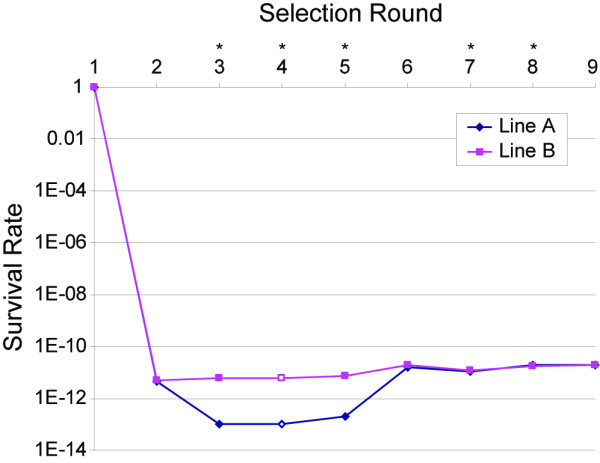
**The survival rate of Line A and Line B isolates throughout extended thermal selection**. This rate was calculated based upon the PFU values at time 0 (ancestral control lines) and the number of PFU after each treatment. The PFU values measured for treatment 4 (open square and triangle for Line A and B respectively) are approximated values due to the less than exact amounts of treated solution available for plating. The asterisks are the treatments in which the phage lysate was collected from the previous treatment's plating and incubation at 37°C for 24 hours.

As expected based upon the short-term heat-treatments, the majority of the replicate lineages did not survive the initial treatment at 70°C; in fact, of the estimated 1 × 10^15 ^phage within the 1 ml tube treated, less than 10^-13 ^survived the first two treatments. For the 18 replicates, only one of each line survived the subsequent eight rounds of selection. Figure 4 shows the relative percentage of surviving phage; denoted by the asterisks are the treatments in which the phage lysate was collected from the previous treatment's plating and incubation at 37°C for 24 hours. As a result of these platings, it is very likely that the volume of phage is not held constant throughout the duration of the thermal treatments. Despite this, two important observations can be drawn from the figure. Firstly, because both lines are undergoing the same treatment regiment after the first treatment, one can compare the survival and relative changes in fitness (in the treatment's ability to infect and replicate during the plating) between the two lines. As shown in the figure, the phage population of Line A decreased after the second selection round whereas the Line B population remained rather static after the initial treatment. After subsequent heating, however, Line A regained fitness and the ability to withstand prolonged heating at 50°C. Both lines showed an improved survival rate at 50°C than they did after the initial long-term exposure to 70°C during the first treatment. Secondly, one can see that the surviving phages (regardless of the line) do not experience a rapid increase in fitness and regain a titer comparable to the initial ancestral line during subsequent temperature treatments.

One of the factors which can affect phage fitness is its ability to be adsorbed by the host species. Thus the adsorption rate for each of the surviving phages (after all nine rounds of selection) was determined to be 1.5 × 10^-9 ^ml/min for Line A and one order of magnitude less, 1.91 × 10^-10 ^ml/min for Line B.

To determine if these isolates were more capable to withstand elevated temperatures than the JACS or Anc strains, five replicates of the surviving lineages (at the same initial titer) were heated for 4 hours at 50°C and 4 hours at 70°C. Both the Line A and Line B isolates treated at 50°C appeared identical to the untreated JACS and Anc strains as well as the treatments from the short-term experiment. In contrast with the results of the short-term exposure to 70°C, Lines A and B appeared identical to the untreated JACS and Anc strains; dilution series were performed showing no detectable decline in titer.

DNA from the two surviving lineages was isolated, amplified, and sequenced (see Methods) in order to uncover which molecular changes were responsible for the isolate's ability to withstand long periods of elevated temperatures. Table 5 lists the mutations identified; Line A includes one nonsynonymous mutation and one synonymous mutation and Line B includes two nonsynonymous mutations. The sequence for Line A has been deposited [GenBank: GU385905]. Interestingly, the mutations within the two surviving lines occurred at positions different from those observed during the sequencing of the short-term heat treatments. Moreover, the mutations which occurred in the acute thermal selection experiments were not seen in the surviving long-term treatment isolates. To assess the uniqueness of the individual mutations within the lines, the isolate's genome was aligned with 66 publically available ΦX174 genomes. The presence of threonine at amino acid position 100 of the F protein is common amongst the sequences (occurring in 58 other strains while the alanine of the ancestor JACS is present in seven other strains). The lysine at amino acid position 151 of the H protein is also common; in fact the JACS ancestor is the only strain with another amino acid at this position.

**Table 5:** Mutations observed in the long-term heat treated isolates.

		Amino Acid Observed		
Genome Position	Coding Region	JACS	Line A	Line B
1301	F	Ala	Thr	Thr
3386	H	Asn	Asn	Lys
4784	A/A*	His	His*	His

* Indicates synonymous mutation

## Discussion

Exposing phage to elevated and extreme temperatures can have one of two principal effects. Firstly, as previous studies have shown, mutations can be induced by heat [15]. Thus new genetic variations can arise within the population as a result of increased temperatures. Secondly, genetic variants present within a population can respond differently to their new environmental conditions. Thus, genotypes with the ability to tolerate this new environment, regardless of their initial frequencies within the population, will survive while others may perish. A combination of the two is also possible.

We can draw two conclusions based upon the results of exposing the phage to acute thermal selection. Firstly, heating the wild-type ΦX174 at 50°C for brief periods of times had no or little effect on phage viability and does not lead to the introduction of genetic variation or increase within the population of genetic variants. No decline in phage titer occurred between one and four hours suggesting that the phage is inherently able to withstand this heat in an aqueous environment, despite the fact that it is well above the temperature that is comfortable for its host. Secondly, exposure to 70°C for even a short period of time greatly diminishes ancestral phage survival, although some phages persist in this harsh environment. The morphology of the plaques isolated after 70°C selection were similar to those produced by the untreated ΦX174, albeit drastically fewer in number.

The fact that the 50°C treatment line sequences and subsequent resequencing of plaques from the control lines do not include the mutations detected within the 70°C treatment sequences suggests that the mutations observed within the lines are in response to their exposure to the elevated temperature rather than possible variation present within the initial phage population. As such, one could conclude that all (or a subset) of the mutations found confer the ability to withstand brief exposure to 70°C. To confirm this hypothesis, these isolates were subjected to another round of heating (for the same duration as previously administered) and replated; the number of PFUs exceeded that of its previous plating (results not shown) indicating that these mutations do in fact correspond with heat resistance.

The mutations detected within the sequences of the heat tolerant plaques reveal that there are multiple mutations which are associated with the response to temperature. Three of the isolates, one treated for 2 hours and two treated for 3 hours, had the exact same set of mutations. All but one of the eight isolates contained the transversion G→ T (Q349H) at nucleotide position 2050 within the F coding region and the transition C→ T (T63S) at nucleotide position 3120 within the H coding region. The missense mutation at the position in F is believed to have a role in stabilizing the viral capsid as well as allow fusion of the viral particle to the bacterial cell membrane [25,26]. Histidine, unlike glutamine, can support cross-linking within the viral capsid by accepting protons from adjacent amino acids, which may provide support and reinforce the viral capsid during the extreme hot temperatures [27]. Furthermore, glutamine residues have been characterized as essential for entry of enveloped viruses into their host cells; without which the viral capsid is unable to fully fuse with the bacterial cell membrane [28]. The function of the H protein or the minor spike protein is attachment of the virion to the host lipopolysaccharides [29]. One may thus speculate that this particular missense mutation may have allowed the isolates to remain viable at extreme temperatures. In a stability experiment, the cysteine amino acids in the spike protein of phage P22 were replaced with serine; the resulting mutant could survive at temperatures of up to 75°C [30]. The combination of the two mutations occurring together suggests epistasis, and that the selective benefit of one of the mutations is contingent on the presence of the other. From these data alone, it is unclear which of the mutations is truly beneficial, and if the selective benefit of the second mutation is due to amelioration of the pleiotropic costs of the first [31].

One of the isolates treated for 3 hours is unique in that a single missense mutation within the H coding region (G79V) not only conferred tolerance to the extreme temperature for 3 hours of exposure but also improved the rate in which the phage could be adsorbed (Table 2); the ability to be adsorbed more quickly, although not specifically selected for here, should lead to higher fitness for the phage mutant strain [24]. This isolate's improved adsorption rate may be attributed to valine's contributor to cell anchoring [32]. This single mutation in fact increases the rate in which it can be adsorbed exceeding that of its ancestor. Additionally, the survival rate of this particular isolate was the highest of any of the short-term treatment lines, 1%. This suggests that the single exchange of a glycine to valine may in fact both increase adsorption and provide resistance to elevated temperatures, at least for a short duration. The phenotypic ramifications of this single mutation warrant further investigation.

**Figure 5:**
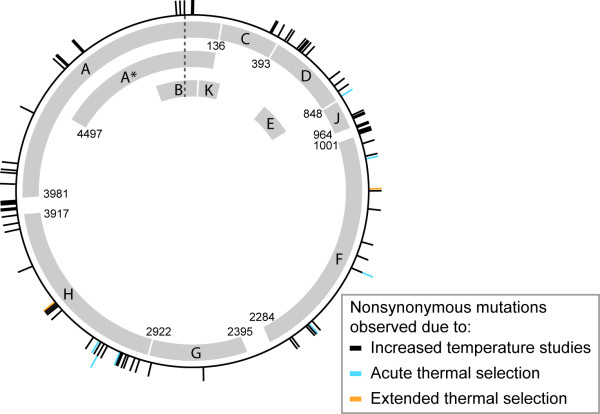
**Nonsynonymous mutations observed in the ΦX174 genome due to elevated temperature conditions**. Those denoted in black are from other studies [16,17,19,20]; those denoted in blue are from the acute thermal selection experiments presented here; and those in gold are from the extended thermal selection isolates.

The C→ T transition at the 1727 nucleotide position within the F coding region (L242F) appears in four heat isolates all of which also include E150D, Q349H, and T63S. This particular transition has been previously identified [16,19,20] (Figure 5); in fact in the introduction of high temperatures (45°C) in the study of Bull *et al*. [16], this particular mutation was recovered in ~85% of the isolates. The mutation found in the study of Bull *et al. *[16] at nucleotide position 1565, however, was not detected within the acute thermal selection experiments conducted here. The other mutation within the F protein found in this study (A45V) has not been previously detected with respect to elevated temperature experiments (Figure 5).

The results of the acute thermal selection experiments may be the result of heat-induced mutation or the result of proliferation of particular genetic variants present within the initial population. As Table 3 shows, several of the different lines independently converged upon the same mutations suggesting that these mutations, conferring the ability to withstand extreme temperatures, may: (1) be preferred sites of heat mutagenesis [15] while (2) maintain the phage's ability to infect and replicate successfully within its host. The question, however, remained: *Would these same mutations afford the phage with resistance to longer periods of elevated and/or extreme temperatures*?

The extended heat selection gave surprising results in comparison to the acute selection. None of the mutations observed in the eight sequenced acute selection isolates were found in either of the two sequenced extended selection isolates. While the acute results indicated that adaptive responses to thermal stress could be observed with sufficiently large populations, they were not predictive of longer-term molecular evolutionary responses. The absence of similar prevalent genotypes between the acute and extended selection treatments and between the two extended selection isolates strongly suggests historical factors played important roles in the molecular outcomes. The historical factors likely involve differences in the initial selective regime, as observed to differences in the chance fixation of adaptive mutants [33]. The acute isolates all experienced relatively short high temperature exposures, four hours or less, while the extended isolates both experienced long initial exposures, 24 hours or more.

As presented here, one can see that the protocol employed for the extended thermal selection experiments (Figure 3, Table 4) differs greatly from that of the acute thermal selection experiments. The fluctuations in duration and temperature reflect our efforts to expose the phage lysate to extreme temperatures while simultaneously avoiding complete annihilation of the population. Moreover, the starting ancestral strain for the extended experiments differs from that used for the acute experiments by two nonsynonymous mutations. This was an unintentional result of creating the high titer phage lysate prior to starting treatments; this was not a factor for the acute thermal experiments but actually does provide insight into how the phage was able to survive extreme temperatures for days.

Firstly, if we look at Line A, we find that only one nonsynonymous mutation separates its ancestor JACS and itself. The Line B survivor also contains this same nonsynonymous mutation Thr100 in the F coding region. The Anc strain, which was used as the ancestral line for the acute thermal selection experiments, also includes Thr100. It is highly unlikely, however, that the acquisition of threonine at this position conferred thermotolerance. This conclusion is supported by the fact that the re-heating of the surviving Line A and B strains for 4 hours at 70°C revealed a significantly greater survival rate than that seen in the acute thermal selection experiments. Following the same rationale, we can hypothesize that the nonsynonymous change N151K within the H coding region of the surviving Line B isolate does not confer its thermotolerance as this same amino acid within the Anc line also includes a lysine at this position.

If we discount the mutations observed within the surviving lines, how can one explain the ability for these lines to survive their extended exposure to extreme temperatures? Returning to the results of the acute thermal selection experiments, we concluded that the exposure to 50°C did not introduce an extreme stress upon the phage, as no decline in the survival rate was detected. Thus, one could conjecture that the mutation(s) which inferred the thermotolerance of Line A and B was lost during the final five treatments at 50°C as the conditions were relaxed. The fact that the number of mutations observed within both Line A and Line B are not unique to the individual isolates indicates that ΦX174 is inherently resistant to elevated temperatures (50°C) even for long periods of time.

Another possibility exists - the presence of the asparagine within the H protein (Asn151) assisted in the phage's ability to withstand the extreme temperatures. Although we know that protein H is present within the external spikes of the viron [34], the atomic structure of protein H is still not resolved; bioinformatic analyses predict an N-terminal transmembrane and several coiled-coil domains [35]. The mutation within the JACS ancestral line and the surviving Line A occurs in the beginning of the first coiled-coil domain [35]. It is predicted that the N-terminal end is coiled in the depression on the external surface of each spike and the remaining coiled-coil domains are positioned within the capsid [36]; upon binding with the host LPS, it has been documented that the H protein changes its conformation [37]. Until the structure is in fact known, however, one can only speculate that the Asn151 aids in the viron's ability to survive extreme temperatures.

Recently, we subjected numerous samples of the Anc high-titer lysate (which is identical to the surviving Line B isolate) to 70°C for 24 hours and were unsuccessful in producing any viable phage. On the contrary, when the phage collected from the surviving Line A were exposed to 24 hours at 70°C and then plated, plaques were observed. The same was found to be true when exposed to 80°C, a temperature more extreme than that considered in the long-term treatment regiment. While not conclusive this suggests that the presence of Asn151 may play a role in the ability to withstand this extreme temperature for longer durations. With regards to the Line B survivor, one could speculate that the nonsynonymous mutation, N151L, occurred once the temperature was reduced to 50°C in rounds 5 through 9 of the treatment protocol (Table 4). Further investigation of the impact of Asn151 is warranted.

## Conclusions

Using the model organism ΦX174, we have been able to study adaptive evolution on the molecular level under extreme thermal changes in the environment, which to-date had yet to be thoroughly examined. Using both short-term and long-term heat treatment protocols, we were able to observe mutations that occurred in response to excessive external pressures independent of concurrently evolving hosts. Acute thermal selection resulted in many nonsynonymous mutations; these mutations very likely occurred out of necessity for survival when the phage is forced to evolve to withstand new environmental conditions. Because of the appearance of several mutations in tandem, it leads us to believe that the combination of mutations, specifically Q349H within the F coding region and T63S within the H coding region, occurred to increase phage resistance to high temperature levels. Interestingly, this pair of mutations was not previously observed together in temperature treatment studies in which both ΦX174 and its *E. coli *host were heated [16,19,20]. Extended thermal selection, however, produced less than certain conclusions. None of the mutations identified in the acute thermal selection experiments appeared in the final sequenced surviving lines. The mutations detected within the final surviving lines, moreover, provided very little insight into how the two lines survived the lengthy exposure to extreme temperatures. The presence of Asn151 within the H protein of initial ancestral strain JACS may potentially be responsible, necessitating further investigation. The results of the acute and extended thermal selection experiments, together, confirm that ΦX174 possesses an inherent means of tolerating elevated temperatures of 50°C.

Most notably, the fact that under either short or long durations of elevated or extreme temperatures, exceeding that previously explored [5,13,14,16,17,19,20], confined solely to the phage rather than the phage-host pair resulted in many mutations which have until now not been associated with ΦX174's ability to withstand increased temperatures. We thus hypothesize that the mutations found by our predecessors [16,17,19,20] which were not observed in our experiments are likely in response to changes within the host induced by the new thermal condition in which the phage-host pair is subjected. Even though its host cannot tolerate extreme temperatures such as the ones tested here, this study confirms that ΦX174 is capable of survival.

## Methods

### Strains and Culture Conditions

The ΦX174 Anc and host *E. coli *C strains were obtained from C. Burch (University of North Carolina, NC). In anticipation that the heat treatments to be administered would kill many of the phages, high titer phage lysates (~1 × 10^15^) were created by successive cycles of plating, harvesting and concentrating (reducing the amount of saline solution in which the harvested phage were suspended) of the ΦX174 Anc strain. Dilution series were performed in duplicate to quantify the increase in titer. This process was carried out twice, once prior to beginning the short-term thermal experiments and again prior to conducting the long-term thermal experiments. These phage strains were sequenced. The phage population within the high-titer solution for the short-term experiments was confirmed to be that of the published strain [GenBank: AF176034] [20]. The genomic sequence determined for the initial high-titer population of the extended thermal selection experiments, however, was a derivative of the Anc strain, which we refer to as JACS and have deposited [GenBank: FJ849058]. The JACS strain differs from AF176034 by just two nonsynonymous mutations (amino acid 100 in the F protein and 151 in the H protein) and emerged while producing a high titer phage stock population. The mutation observed within the F coding region is present in other sequenced ΦX174 genome sequences although the asparagine replacement of lysine does not appear in any of the published sequences.

Phage isolates were plated as follows: 100 μl of phage lysate was added to 3 ml 0.5% agar LB and 1 ml of *E. coli *C culture and then overlaid on a 1.7% agar LB plate. Plates were incubated overnight at 37°C. Fresh cultures of *E. coli *C were made daily from the previous day's *E. coli *C culture. All of the *E. coli *C cultures were incubated within a culture tube and isolated from cultures containing phage in an effort to eliminate the possibility of contaminating the host culture and the potential acquisition of phage resistance. Thus we were able to maintain a naïve population of the host. The following ratio was utilized to generate fresh populations of *E. coli *C: 1 ml of the previous day's *E. coli *C culture to 3 ml of LB. Using this ratio, we were able to obtain a reproducible turbid culture within 90 minutes. In an effort to maintain a static genotype and phenotype of the host species, the *E. coli *C liquid culture was plated every fifth day such that the subsequent day's *E. coli *C was derived from a plated colony rather than the preceding day's liquid culture. Plates were harvested and suspended in 0.8% saline solution and treated with 50 μl chloroform. Viral lysates were stored both at -80°C in 50/50 glycerol/water (v/v) as well as at 4°C.

### Short-term heat treatment

To assess the thermotolerance of the ΦX174 bacteriophage, the Anc high titer phage lysates (~1 × 10^15^) were treated at either 50°C or 70°C for 1, 2, 3 or 4 hours and subsequently plated with its native *E. coli *C host. Figure 1 depicts the heat scheme employed. 110 μl wild-type ΦX174 suspended in 0.8% saline solution was aliquoted into individual microcentrifuge tubes and heated using a digital dry heat bath at either 50°C or 70°C for 1, 2, 3 or 4 hours. The wells of the bath were filled with water prior to beginning the heat treatment and the tubes were submerged to ensure a constant temperature surrounding them. Each temperature and duration was performed in triplicate. Following heat exposure, the tube was incubated for one hour at 4°C and then plated following the protocol detailed above. Two 110 μl of the wild-type ΦX174 were also plated to serve as a control. The control line provided a means of determining the expected number of plaque forming units (PFUs) expected when no treatment was applied. The control line was taken from the same lysate as the individual lines which were subjected to heat treatment. Thus, the plating of the control is the survival rate for all of the experimental lines of the high titer phage lysate at time zero.

### Long-term heat treatment

1 ml of the high titer ΦX174 JACS strain suspended in 0.8% saline solution was subjected to a series of heat treatments sequentially shown in Table 4. After each round of selection, 100 μl was extracted and plated as described above; after overnight incubation at 37°C, the plate was assessed with regards to the number of PFUs and individual plaque size. These plates were then subsequently harvested and stored both at -80°C and 4°C as detailed above. Intermittently, the entire phage population was plated as described above; after overnight incubation at 37°C, the plate was harvested and suspended in 0.8% saline solution and used for subsequent heat treatments. The processes of replating the entire treatment sample was conducted as a measure of control given the fact that the number of viable phage was reducing which in turn increased the probability that the randomly selected 100 μl aliquot collected would not contain any viable phage. Similar to the short-term heat treatments, the individual microcentrifuge tubes were submerged within the wells of a dry bath in water (Figure 3). Six replicates were done initially, followed by an additional twelve replicates subjected to the same protocol as described in Table 4.

Two lines, distinguishable by the duration of this first treatment, were introduced in an effort to explore the heat-shock tolerance of a naïve phage; moreover, separation of these two lines allows for the examination of the impact of this initial treatment on subsequent tolerance of elevated temperatures. The dramatic increase in the duration of the temperature exposure, in comparison to that of the short-term treatment experiments, was selected as a result of earlier testing in which intermediary times were explored (results not shown). As treatment time approached 24 hours, we observed minimal survival and thus chose to expand the short-term study from hours to days. Moreover, more uniform (with respect to time and temperature) cycles were explored but without success (results not shown).

When the treated lysate plated produced very few and/or abnormally small plaques, one of two courses of action were followed: the temperature was reduced or the duration of treatment was reduced. This allowed us to more closely monitor the progress of the treatment and avoid inadvertently killing off the phage. It is important to note, however, that both lines were subjected to the same treatment regiment post the initial treatment; in other words, if the plating of Line A appeared normal while Line B did not, the next round of selection for both lines was adjusted.

In the event that upon replating the entire treatment tube the replicate line appeared not to contain any viable phage, the plate was still harvested and treatment was continued through the next replating step. In the case that once again the lineage did not produce any visible plaques, the lysate was stored at 4°C and excluded from subsequent treatments. Upon the conclusion of the series of treatments all such lineages were again replated verifying the absence of viable phage (results not shown). Plating was not performed after every treatment in an effort to minimize the exposure to relaxed selection. These periods of relaxed conditions (24 hours at 37°C), however, are vital in order for the phage to reproduce as well as in providing an opportunity for the phage to introduce genetic variation. It is worth noting, however, that genetic variation is in fact being introduced despite the absence of the host as the extreme temperatures can, in fact, introduce mutations in the absence of the host [14,15].

### Survival Assays

10 μl of the treated lysate was added to 1 ml of 0.8% saline solution and mixed. 10 μl of this mixture was then added to new tube of 1 ml saline solution. This titration was repeated to produce a 1:100 dilution series. One of two strategies was followed in order to quantify the number of phage present in the sample: (1) 100 μl of saline suspended phage lysate from each titration was added to 1 ml of turbid *E. coli *C culture (grown in the same manner as previously discussed) and 3 ml 0.5% agar LB and plated on a 1.7% agar LB plate or (2) 10 μl from each titration was then spotted onto a plate with an *E. coli *C lawn (3 ml 0.5% agar LB and 1 ml of turbid *E. coli *C culture plated on a 1.7% agar LB plate and dried near an open flame). Pictures of each plate were taken using the UVP ColonyCounter (Upland, CA). The number of distinct and isolated plaques on each plate were counted both manually and using the ColonyCounter software. The number of phage present in each isolate was then calculated according to the titration and plating strategy used. In a similar manner, the number of PFU for the control (untreated) phage lysate samples was determined. These control lines serve as time point 0 prior to treatment. The ancestor of the 24 individual treatment lysates, was plated in duplicate for the short-term treatment. Likewise, the ancestral strain of the long-term treatment lysates, the JACS strain, was also plated. In order to ascertain the survival rate of each individual phage treatment or lineage, the treated phage's number of PFU was divided by its respective control line's number of PFU.

### Adsorption Rate Assays

To evaluate the growth rate of the ancestral strains and heat treated lineages, an adsorption assay was performed. The assay estimates fitness based on the doublings of phage concentration per hour, a factor which is not scaled to generation time which may differ among the phages. This allows for a comparison between the ancestor strain and treated strains based on their absolute growth rate with their native host *E. coli *C. The assay is an additional measure of fitness and determines which phage can grow the fastest. The methodology employed was identical to that detailed previously [24]. In summary, *E. coli *C was grown for 90 minutes until visible turbidity was observed. 10 ml of *E. coli *C was then placed into a 13 mm culture tube with 1 ml of bacteriophage and incubated at 37°C. After 5 minutes, 1 ml of the culture was removed and the phage within the supernatant was isolated and titered. This represents the initial concentration of phage (*N*_*o*_). After 60 minutes (*t*), another 1 ml of the culture was removed and the phage in the supernatant was again isolated and titered. This is considered the final concentration of phage (*N*_*t*_). To find the adsorption rate (*k*), the equation *N*_*t *_= *N*_0_*e*^-*kCt *^can be used where *C *is the bacterial cell density [24]. The cell density was kept approximately constant between assays (10^8^/ml) by beginning the assay with the same frozen bacterial stock.

### Sequencing

Genomic extraction was performed using UltraClean™ Microbial DNA Isolation Kit following the standard protocol with an additional heating of the prep for 10 minutes at 70°C to increase lysis efficiency (as suggested by protocol) (Mo Bio Laboratories, Inc., Carlsbad, CA). For the long-term heat isolates, the resultant DNA was used as PCR template and amplified by twelve pairs of PCR primers using the Platinum Taq kit (Invitrogen, Carlsbad, CA). This set of primers were designed in our laboratory using the Primer3 web-application [38] ensuring a minimum 2× coverage of the genome (primers available upon request). For the short-term heat isolates, two pairs of the primer set were used to specifically amplify the region coding for the structural proteins, D, F, G, H and J, with 2× coverage. The reason that this particular portion of the genome was of interest is that the most frequently observed mutations previously observed within ΦX174 strains exposed to elevated temperatures occurred within the structural proteins, particularly F, G and J [14,16,19]. PCR products were purified using ExoSAP-It (US Biological, Swampscott, MA) and sequenced by the University of Chicago Cancer Research Center DNA Sequencing Facility (Chicago, IL).

### Sequence Analysis

The sequences generated in this study were assembled using LaserGene SeqMan (DNASTAR, Inc., Madison, WI). Comparisons between the isolate contigs to the ancestral strain's sequence were conducted using the ClustalW application within BioEdit [39]. Other ΦX174 genome sequences were collected from NCBI by following the "Other genomes for species" link on the RefSeq NC_001422 overview. The ClustalW application within the BioEdit application [39] was used to align the 66 complete genomes gathered from NCBI with the genomes assembled in this study. In order to identify the point mutations previously attributed to elevated temperature, the complete nucleotide sequences were downloaded from the study of Crill *et al. *[20], which includes the ancestral strain Anc of the short-term heat experiments, [GenBank: AF176027- AF176034]. Alignments were performed as before and all unconserved positions were identified. Figure 5 reflects these mutations as well as others collected from the literature [16,17,19].

## Authors' contributions

CP and MT conceived and designed the study. JC and AS performed all of the experiments. JC, AS and CP performed the analysis. All authors participated in writing the manuscript.

## Supplementary Material

Additional file 1 **Nucleotide changes observed in the short-term heat (70°C) treated isolates**. Nucleotide changes occurring within short-term treatment lineages listed in Table 3. Click here for file
