# Supplementary material for: Adaptive evolution and inherent tolerance to extreme thermal environments
Source: BMC Evol Biol. 2010 Mar 12;10:75. doi: 10.1186/1471-2148-10-75 (PMC2850354; doi:10.1186/1471-2148-10-75)
Supplement: Additional file 1 — Nucleotide changes observed in the short-term heat (70°C) treated isolates. Nucleotide changes occurring within short-term treatment lineages listed in Table 3. [file 1471-2148-10-75-S1.PDF]

Duration of Selection: 1 HOUR
E150D: Nucleotide genomic position 840 changed from a G to a T
L242F: Nucleotide genomic position 1727 changed from a C to a T
Q349H: Nucleotide genomic position 2050 changed from a G to a T
V44L: Nucleotide genomic position 3063 changed from a G to a T
T63S: Nucleotide genomic position 3120 changed from a C to a T

Duration of Selection: 1 HOUR
Q349H: Nucleotide genomic position 2050 changed from a G to a T
T63S: Nucleotide genomic position 3120 changed from a C to a T

Duration of Selection: 2 HOURS
E150D: Nucleotide genomic position 840 changed from a G to a T
A45V: Nucleotide genomic position 1137 changed from a C to a T
L242F: Nucleotide genomic position 1727 changed from a C to a T
Q349H: Nucleotide genomic position 2050 changed from a G to a T
T63S: Nucleotide genomic position 3120 changed from a C to a T

Duration of Selection: 2 HOURS
Q349H: Nucleotide genomic position 2050 changed from a G to a T
T63S: Nucleotide genomic position 3120 changed from a C to a T

Duration of Selection: 3 HOURS
G79V: Nucleotide genomic position 3169 changed from a G to a T

Duration of Selection: 3 HOURS
E150D: Nucleotide genomic position 840 changed from a G to a T
A45V: Nucleotide genomic position 1137 changed from a C to a T
L242F: Nucleotide genomic position 1727 changed from a C to a T
Q349H: Nucleotide genomic position 2050 changed from a G to a T
T63S: Nucleotide genomic position 3120 changed from a C to a T

Duration of Selection: 3 HOURS
E150D: Nucleotide genomic position 840 changed from a G to a T
A45V: Nucleotide genomic position 1137 changed from a C to a T
L242F: Nucleotide genomic position 1727 changed from a C to a T
Q349H: Nucleotide genomic position 2050 changed from a G to a T
T63S: Nucleotide genomic position 3120 changed from a C to a T

Duration of Selection: 4 HOURS
Q349H: Nucleotide genomic position 2050 changed from a G to a T
T63S: Nucleotide genomic position 3120 changed from a C to a T

Supplemental Table S1. Nucleotide changes occurring within short-term treatment lineages listed in Table 3.
